# Analysis of the relationship between end-to-end distance and activity of single-chain antibody against colorectal carcinoma

**DOI:** 10.1186/1742-4682-9-38

**Published:** 2012-08-22

**Authors:** Jianhua Zhang, Shanhong Liu, Zhigang Shang, Li Shi, Jun Yun

**Affiliations:** 1 Faculty of Biomedical Engineering of Zhengzhou University, Zhengzhou, 450001, Henan Province, People's Republic of China; 2 Emergency Department of Xi’an North Hospital, Xi'an, 710000, Shaanxi Province, People's Republic of China; 3 Vascular Endocrinology Department of Xijing Hospital Affiliated to the Fourth Military Medical University, Xi'an, 710032, Shaanxi Province, People's Republic of China

**Keywords:** Single-chain Fv antibody (scFv), (Gly_4_Ser)n, End-to-end distance, Homologous modeling, Meta MQAP

## Abstract

We investigated the relationship of End-to-end distance between VH and VL with different peptide linkers and the activity of single-chain antibodies by computer-aided simulation. First, we developed (G_4_S)_n_ (where n = 1-9) as the linker to connect VH and VL, and estimated the 3D structure of single-chain Fv antibody (scFv) by homologous modeling. After molecular models were evaluated and optimized, the coordinate system of every protein was built and unified into one coordinate system, and End-to-end distances calculated using 3D space coordinates. After expression and purification of scFv-n with (G_4_S)n as n = 1, 3, 5, 7 or 9, the immunoreactivity of purified ND-1 scFv-*n* was determined by ELISA. A multi-factorial relationship model was employed to analyze the structural factors affecting scFv: $r\left(n\right)=\sqrt{{\left[AB\left(n\right)-AB_{O}\right]}^{2}+{\left[CD\left(n\right)-CD_{O}\right]}^{2}}+{\left[BC\left(n\right)-BC_{\mathrm{st}}\right]}^{2}$. The relationship between immunoreactivity and *r*-values revealed that fusion protein structure approached the desired state when the *r*-value = 3. The immunoreactivity declined as the *r*-value increased, but when the *r*-value exceeded a certain threshold, it stabilized. We used a linear relationship to analyze structural factors affecting scFv immunoreactivity.

## Introduction

Single-chain Fv antibody (scFv) is composed of immunoglobulin heavy- and light-chain variable regions connected by a short peptide linker [1-3]. ScFv is an ideal tool for the construction of single-chain bi-specific antibody fusion proteins [4-6]. Bivalent antibodies derived from scFv using genetic engineering have a promising future in the clinic. scFvs can be therapeutic and at the same time serve as a vector for delivering a toxin [7]. In recent years, there has been progress in colorectal cancer diagnosis and treatment using scFv as a carrier. However, achieving both high affinity and anti-tumor activity can be difficult, particularly since both are needed to be effective. Studies have shown that a proper linker can provide a scFv with biological activity more effective for clinical applications [8-10]. Consequently, choosing and designing a proper linker is a key consideration.

Proteomics has revealed a great deal about the composition, structure, and function of proteins, and bioinformatics provides a powerful tool to study the structure-activity relationship of fusion proteins [11-13]. Drug design based on structural simulation incorporates 3D structure, including data from fusion proteins with various functional domains and inter-peptide linkers [14-16]. Linkers that contain (G_4_S)n are the most widely used [12,17], prompting us to examine its effects on the structure and function of scFvs.

## Materials and methods

### Materials

IC-2 and CCL-187 cells were cultured using standard conditions. IC-2 is a murine hybridism cell line that secretes the monoclonal antibody ND-1, specific for human colorectal carcinoma. CCL-187 is a human colorectal carcinoma cell line. The pET28a (+) expression vector and *E. coli* BL21 were contributed by Prof. J. Yun, Xi'an (China). The pMD18-T vector, *E.coli* JM109 competent cells, DNA polymerase, restriction enzymes, and DNA recovery kits were purchased from TaKaRa Biotechnology (Shanghai, China). mRNA purification kits and T4 DNA ligase were purchased from Pharmacia Biotech (Shanghai, China). Anti-His6 tag antibody was obtained from Invitrogen (Foster City, CA, USA). Ni-NTA resin was provided by QIAGEN (Shanghai, China), MDP and 99mTc were kindly provided by the Department of Nuclear Medicine of China Medical University (Liaoning Province, China). Heavy chain primer 1 and 2, light chain primer mix, linkers [(GGGGS)n] primer mix, and RS primer mix were purchased from Pharmacia Biotech.

ND-1 scFv-n was constructed as previously described. Briefly, mRNA was extracted from 5 × 10^6^ IC-2 hybridism cells and cDNA synthesized by reverse transcription using random primers. VH and VL genes were separately amplified from cDNA by PCR using a heavy and light chain primer mix. The VH and VL gene fragments were recovered and mixed in equimolar ratios for two PCR reactions, with the first one using a linker primer mix for 7 cycles, followed by a second one using a RS primer mix for 30 cycles. As a result, VH and VL gene fragments were linked to form a scFv construct by extension, with overlapping splicing PCR. The resulting ND-1 scFv-n construct was cloned into pMD18-T and transformed into *E. coli* JM109, and positive clones identified by colony PCR and DNA sequencing.

Oligonucleotide primers S1 and S2 were designed to add *EcoR*I sites at the 5'-end of ND-1scFv-n, and a *Hind*III site, or *SalI* site at the 3'-end. S1: 5'-CTGAATTCATGGCCCAGGTGCAGCTGCAGC-3'; S2: 5'-CGCAAGCTTCTAGTCGACTTTCCAGCTTGGTC-3'. pMD18-T-ND-1scFv-n was used as a template, and the product cloned into the vector pET28a(+) after digestion with *EcoR*Iand *Hind*III, and transformed into competent *E.coli* BL21 cells for protein expression.

### Amino acid sequence

**Figure 1:**
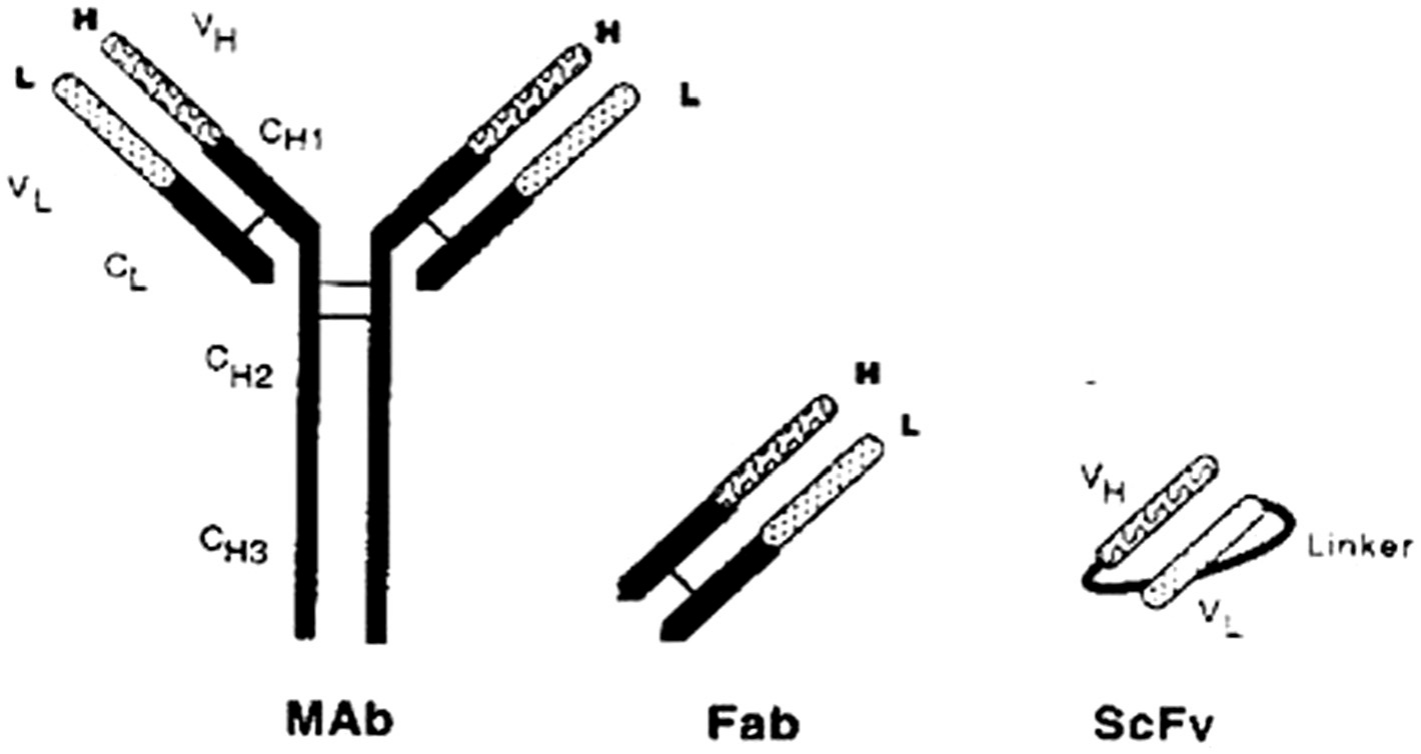
Map of VH-linker-VL.

The amino acid sequence of the wild-type VH and wild-type VL are listed below [18], and illustrated in Figure 1. The amino acid sequence of the VH-(G_4_S)n-VL is:

MAQVQLQQSGPGLVAPSQSLSITCTVSGFSLTTYDVHWVRQPPRKGLEWLGLVW

ANGRTNCTSALMSRISITRDTSKNQVFLTMNSLQTDDTAMYYCARGSYGAVDFWG

QGTTVTVSS(GGGGS)nDIELTQSPASLAVSLGQRATISYRASKSVSTSGYSYMHWQQ

KPGQPPRLLIYLVSNLESGVPARFSGSGSGTDFTLNIHPVEEEDAATYYCQHIRELTRSEGGPSWK.

### Homology modeling, assessment, and optimization

The amino acid sequence of a protein determines its high-level structure. Determining high-level protein structure relies on the identification of one or more known protein "templates" that resemble the structure of the query sequence, and alignment of the query sequence residues to the template residues. Swiss-Models can be used for homology modeling to search protein sequence and structure databases, such as the Protein Data Bank (PDB) [19-21]. A three-dimensional model of the targeted molecule can be obtained through homology modeling, and used to assess and optimize the model using Meta MQAP [22,23].

### Construction of coordinate system

PDB files were obtained from Swiss-Model with the videotext coordinate system (in which the atomic coordinates are located), in order to facilitate protein structure comparison. The coordinate systems were constructed with Matlab7.0.

### Determination of the origin of the coordinate system

The molecular weight of the atoms in the protein was used to calculate molecular weight, and the centric was obtained using the atomic location of each atom. The centric is the origin of the new coordinate system [24].

(1) $$\left[X_{0},Y_{0},Z_{0}\right]=\left[\frac{1}{N}\sum_{k=1}^{N}\frac{X_{k}*m_{k}}{M},\frac{1}{N}\sum_{k=1}^{N}\frac{Y_{k}*m_{k}}{M},\frac{1}{N}\sum_{k=1}^{N}\frac{Z_{k}*m_{k}}{M}\right]$$

N: the number of all the atoms; M = 12.01 + 14.01 + 16.00 + 32.07 + 1.00;

Mi: molecular weight of atoms;

[X_k_, Y_k_, Z_k_]: the original three-dimensional coordinates of atoms;

[X_0_, Y_0_, Z_0_]: the origin of the new coordinate system.

To determine axes we constructed a second-order moment matrix of the protein’s atomic coordinates. This was regarded as the principal component of the matrix’s eigenvector of the new coordinate system’s X-axis, the sub-principal component of the vector Y-axis, and used to build a coordinate system of the protein’s three-dimensional structure.

The 3 × 3 matrix constructed by the second-order moment matrix is as follows:

(2) $$S=\left[\begin{array}{ccc} M_{200} & M_{110} & M_{101}\\ M_{110} & M_{020} & M_{011}\\ M_{101} & M_{011} & M_{002} \end{array}\right]$$

Here, $M_{\mathrm{abc}}=\sum_{k}m_{k}\times X_{k}^{a}\times Y_{k}^{b}\times Z_{k}^{c}\text{,}$

m_k_: molecule weight of atoms.

[X_k_, Y_k_, Z_k_]: 3D coordinates of each atom.

The eigenvalues and eigenvectors of *S* were calculated, and the eigenvector calculated corresponding to the maximum eigenvalue as the first axis (X axis is set, X = [X1, *X*2, X3]), with the eigenvector corresponding to the second largest eigenvalue as the second axis (Y axis set, Y = [Y1, Y2, Y3]), and similarly for the Z axis.

### Analysis of End-to-end distance in fusion proteins

The End-to-end distance is the distance between the first and the last α-carbon atom in a protein. We obtained this information and the X/Y/Z coordinates of the atoms from the PDB database. The algorithm used is as follows:

A. Locate the first and last α-carbon atoms in the wild-type VH and VL, and the same in the protein after introduction of (G_4_S)n.

B. Calculate End-to-end distance of wild-type VH (VL) and mutant VH (VL) after introduction of (G_4_S)n.

C. Analyze the relationship between the End-to-end distance and *n*.

### Biological experiments

Expression and purification of ND-1scFv-*n*.

pET28a(+)-ND-1scFv-n plasmids were constructed as expression vectors and transformed into *E. coli* BL21 cells, which were grown in 100 ml LB broth with 50 mg/ml Kanamycin at 37°C. When the culture attained an O.D. of 0.6, IPTG was added to a final concentration of 1 mM, and cells were shaken at 37°C. After 3.5 h, the culture was centrifuged at 5,000 rpm for 10 min, and the cell pellets treated with lysis solution. After sonication and centrifugation, inclusion bodies containing scFv proteins were solubilized and denatured in the presence of 6 M guanidine hydrochloride. Affinity chromatography on Ni-NTA resin was use to purify scFv, and the column eluted sequentially with 8 M urea at pH8.0, 6.5 and 4.2. The pH4.2 fraction, containing scFv, was collected and recaptured by dialysis. Protein purity and concentration were determined by Bradford assay.

### Western blot analysis

ND-1scFv-*n* proteins were detected by western blot analysis. BL21 transformed with pET-28a(+)ND-1scFv-*n* was incubated separately in loading buffer (125 mmol/L Tris–HCl, pH 6.8, 10% β-mercapto-ethanol, 4.6% SDS, 20% glycerol and 0.003% bromophenol blue) for 5 min at 100°C, separated by sodium dodecyl sulfate polyacrylamide gel (SDS-PAGE), and electro blotted onto PVDF membrane (Bio-Rad, Hercules, CA, USA). Non-specific binding sites were blocked for 1 h with 5% nonfat milk in TPBS (PBS contained 0.05% Twin 20), and the membrane incubated overnight at 4°C with primary antibody. After washing 3X in TPBS, the membrane was incubated with horseradish peroxidase-conjugated goat anti-rabbit IgG for 2 h at room temperature, and washed 2X with TPBS. Immunoblot signal was detected by autoradiography using an enhanced chemiluminescence detection kit.

### ELISA assay for activity of ND-1scFv-n

CCL-187 cells (5 × 10^4^) were grown in 96-well micro titer plates at 37°C for 24 h, fixed with 2.5% glutaraldehyde and blocked with 1% BSA, followed by incubation with ND-1IgG or ND-1scFv at 37°C for 2 h. After washing 3X with PBS, anti-His6 antibody was added to wells with ND-1scFv-n and incubated. The plate was washed and HRP-labeled goat anti-mouse IgG was added into both ND-IgG and ND-1scFv wells. After incubating at 37°C for 2 h, TMB substrate was added, and samples incubated in darkness for 30 min. The reaction was terminated with 1 M H_2_SO_4_. PBS was used as a negative control.

## Results

### Protein structures

**Figure 2:**
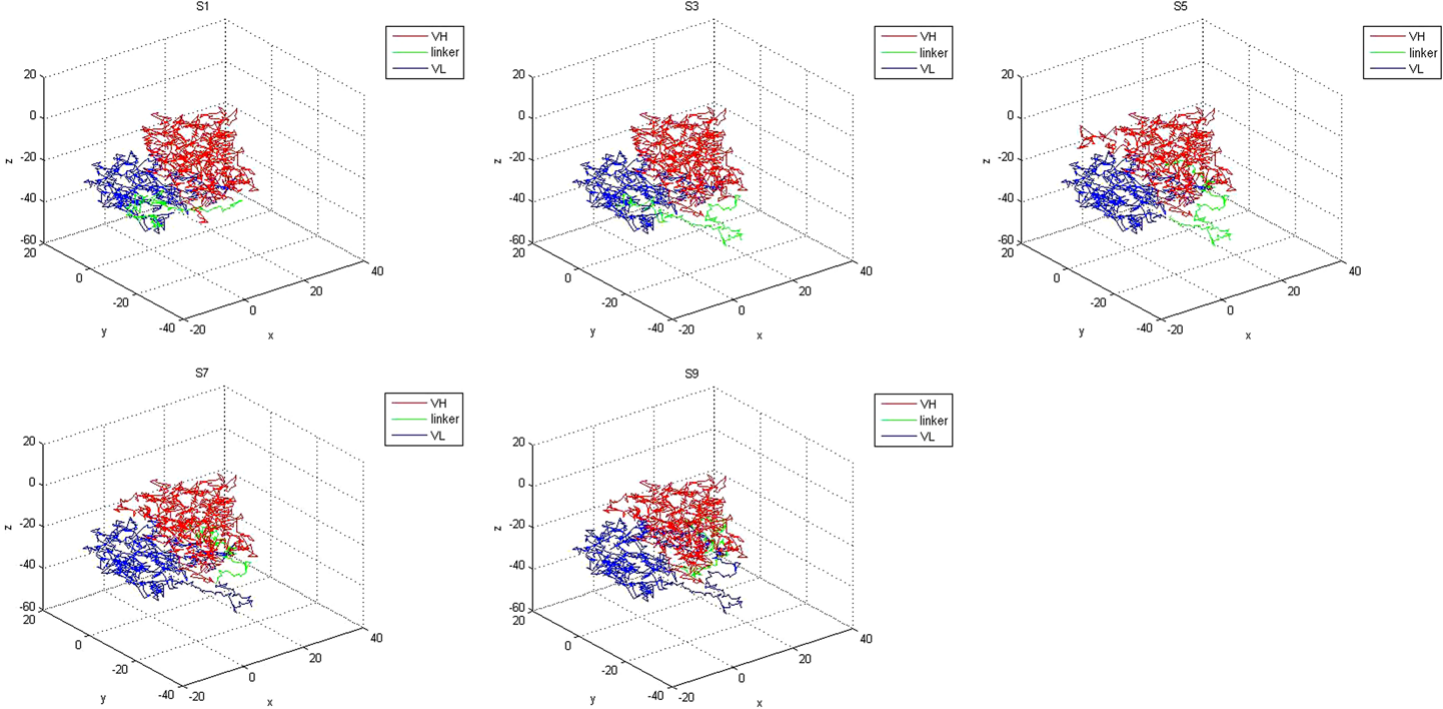
**Map of VH-(G**_**4**_**S)n-VL (LL: linker length; red line: VH peptide; green line: linker peptide; blue line: VL peptide).**

A videotext of the coordinate system was built using the PDB atomic coordinates from PDB files received from SWISS-MODEL, using Mat lab 7.0. The maps were used for comparison of the protein structures (Figure 2). Homology modeling using SWISS-MODEL was used to evaluate the best evaluation method. Meta-MQAP was used to assess and optimize the model. The accuracy score of the model and the root mean square (RMS) deviation are shown in Table 1. The assessment result shows that the model is reliable.

**Table 1:** Global model accuracy was analyzed by Meta-MQAP

**Sequence**	**GDT-TS**	**RMSD**
VHL	64.189	2.392
VHL1	75.548	1.959
VHL2	74.893	1.920
VHL3	64.811	2.400
VHL4	75.332	2.082
VHL5	77.544	2.016
VHL6	75.000	2.120
VHL7	76.991	1.904
VHL8	73.783	2.229
VHL9	60.841	2.860

### Local alignment

**Figure 3:**
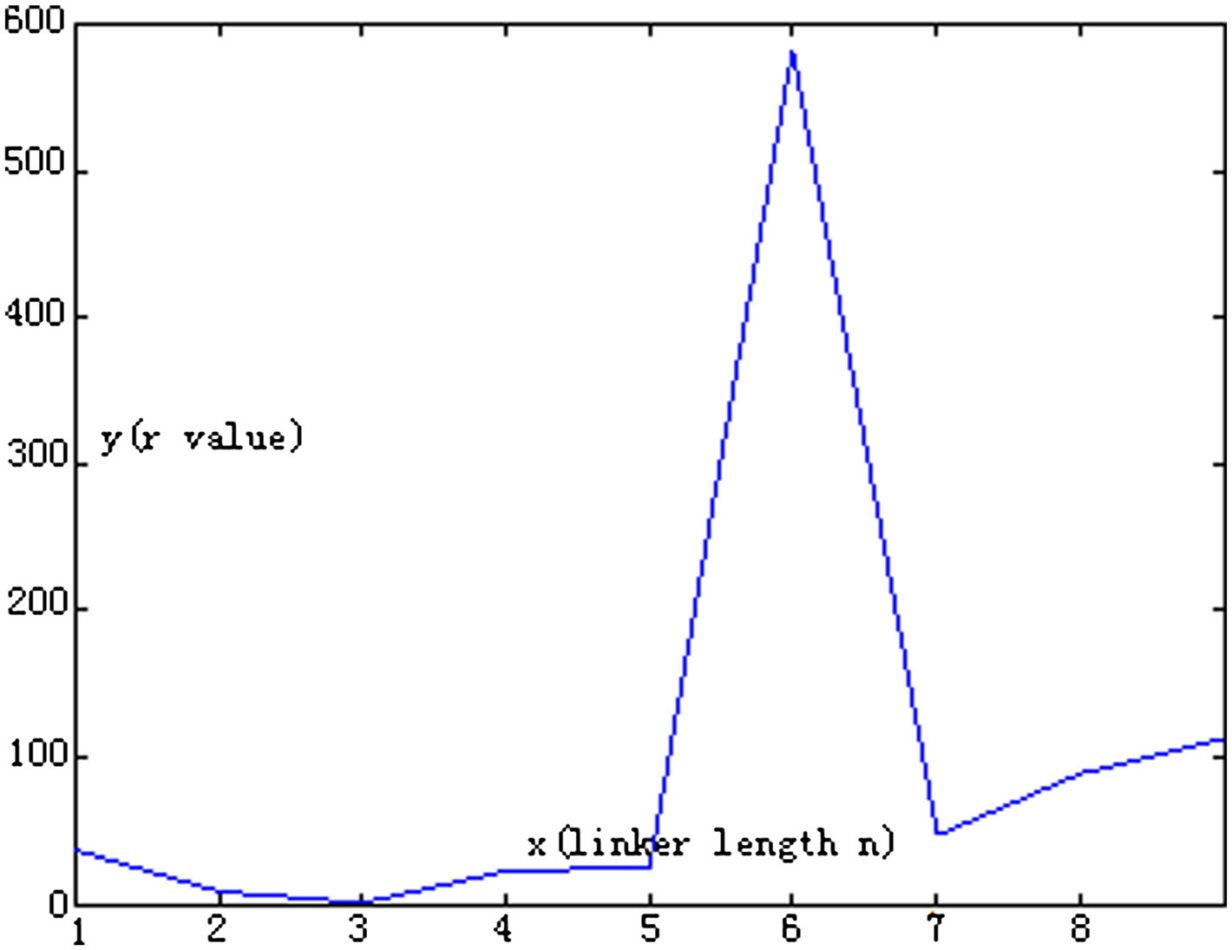
**The diagram shows the relationship between the length of linked peptides and *r *value.**

The End-to-end distance of VH (AB), VL (CD) and linker (BC), at different *n* values are presented in Table 2. It appears that linker BC was relatively stable from *n* = 1-7, and there were changes in the End-to-end distances for AB and CD. When the *n* value increased within a certain range, the End-to-end distance of VH had relatively large fluctuations. The End-to-end distance of VL basically did not change except when *n* = 6 and *n* = 0. The data suggests that the major factor for this was that the median value of BC was about 22.6622 in the End-to-end distances of linked peptides. Although the End-to-end distance changes were small, there were fluctuations in the value of AB and CD near the ideal state. Thus, the effects of the linked peptide structural factors (*r*) on VH and VL can be represented in the following equation: $r\left(n\right)=\sqrt{{\left[AB\left(n\right)-AB_{O}\right]}^{2}+{\left[CD\left(n\right)-CD_{O}\right]}^{2}}+{\left[BC\left(n\right)-BC_{\mathrm{st}}\right]}^{2}$.The ideal fusion protein structure should have a stable structure with the linker peptide of $\min r\left(n\right)$, as shown in Figure 3. The results of *r* were obtained from the corresponding linker length. The *r*-values were 36.8161, 8.0150, 0.8415, 22.1579, 24.4747, 582.2451, 46.8344, 88.6852, and 112.3846, with a median value of 24.4747.

**Table 2:** **The value of AB\CD\BC at different values of *n***

**Sequence**	**BC**	**AB**	**CD**
Wild-type VH	36.7939		
Wild-type VL	14.4022		
n = 0	29.6393	6.8006	
n = 1	16.7822	34.9540	13.1215
n = 2	22.6653	44.7992	14.0089
n = 3	22.6622	37.5364	14.0063
n = 4	19.6630	23.6371	14.0095
n = 5	23.7909	13.59664	14.0052
n = 6	46.6356	37.536	21.8867
n = 7	27.4472	12.8588	14.0167
n = 8	13.9244	24.4632	14.0393
n = 9	12.5718	26.2316	14.0417

The results suggest that when *n* = 3, the *r*-value was the smallest, and the structure of fusion proteins was closest to the ideal state. The *r*-values increased when *n* increased and hence the linker length increased, in which VH and VL structure would be impacted to a greater extent. When *n* was 6, the *r* value was the most unsatisfactory.

### Determination of expression and purity of proteins

**Figure 4:**
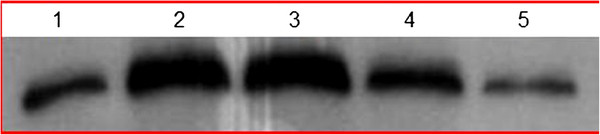
**Western blot analysis of ND-1scFv-n in BL21 cells.** 1: Expression of pET28a (+)-ND-1scFv with (G_4_S)_1_; 2: Expression of pET28a (+)-ND-1scFv with (G_4_S)_3_; 3: Expression of pET28a (+)-ND-1scFv with (G_4_S)_5_; 4: Expression of pET28a (+)-ND-1scFv with (G_4_S)_7_; 5: Expression of pET28a (+)-ND-1scFv with (G_4_S)_9_.

Plasmids ND-1scFv-pET28a (+) were transformed into *E. coli* BL21, and protein expression induced with IPTG. Western blot analysis indicated that BL21 lysates expressed scFv-n proteins with bands of 30 kDa (Figure 4). The sequences encoding the short His-tag peptide were upstream of the multi-cloning site (MCS) of vector pET28a (+), and ND-1scFv-n was expressed as a recombinant fusion protein. Western blot analysis showed that scFv-n protein is expressed in inclusion bodies in the supernatant of BL21 lysates. Inclusion body protein was purified to 94% by metal affinity chromatography using Ni-NTA resin, which binds to the His-tag protein marker on the N terminal end of scFv.

### Analysis of the relationship between immunoreactivity and End-to-end distance

The immunoreactivity of purified ND-1scFv-n was determined by ELISA. scFv-n exhibits an immunoreactivity similar to the parental ND-1 antibody, and demonstrated good binding to CCL-187 cells expressing colorectal carcinoma associated antigen LEA. This suggests that scFv-n retains good specificity and activity.

**Figure 5:**
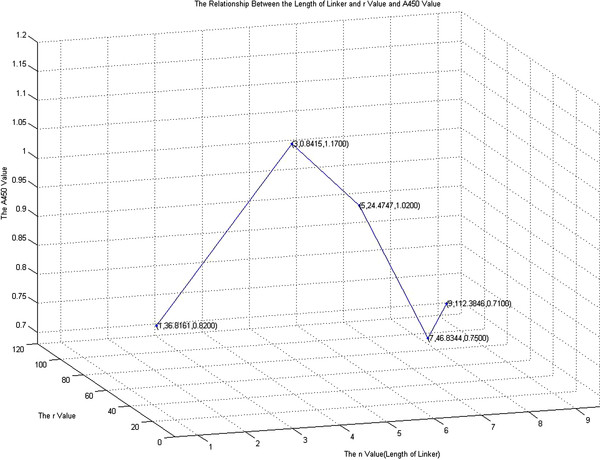
**The linear relationship between the *r *value and ND-lsc(Fv)**_**2**_**-n immune reactivity.**

Table 3 shows the relationship between scFv immunoreactivity (A_450_ value) and *r-values*. The immunoreactivity declined with increasing *r*-values. It changed significantly when the *r*-value was less than 42.3716. When the *r*-value exceeded this value, immunoreactivity became relatively stable (Figure 5).

**Table 3:** **The relationship between *r*-value and the corresponding biology**

**n**	***r *value**	**A450 value**
3	0.8415	1.17
5	24.4747	1.02
1	36.8161	0.82
7	46.8344	0.75
9	112.3846	0.71

## Discussion

Homology modeling has been successfully applied to interpreting the correlation of protein sequence, structure, and function. Using a structural model, multiple sequences of orthologues proteins can be compared and evaluated according to the restrictions of natural selection and requirements of protein folding, stability, dynamics, and function. Homology modeling can help determine which functional groups the protein belongs to based on the analyses of conserved residues in the binding site. Homology modeling also plays an important role in computer-aided drug design [25,26].

One basic issue in the study of protein structure is structural comparison. The relatively direct comparison method is to consider the protein as a rigid structure composed of a series of point sets, then compare the corresponding residues of different proteins. At the beginning, a rigid superposing method was used (to translate and rotate the spatial structure of the protein to find the corresponding residues between two proteins) [27,28]. However, Chen proposed using a weight distribution of the atoms composing the protein, and to use this to calculate the protein’s gravity center, using a 3 × 3 matrix composed of second-order moments [24]. On this basis, one can use principal component analysis (PCA) to find the main and secondary axis. The best rigid superposition is obtained through superposing the gravity centers of the proteins and then rotating them to let their main axes superimpose. In this study, we used the molecular weight of the atoms to get the centric according to the coordinates of each atom.

It is recognized that fusion proteins have varied affinity and anti-tumor activity compared to the original molecules, due in large part to the structural alterations of the fusion proteins [4,28-31]. The inter-peptide linkers can be optimized with computer-aided design [32]. Based on homology modeling of derivatives [33], future designs of inter-peptide linkers can be viewed as solving an equation. The structure and characteristics of target molecules, and the composition, length, and flexibility of inter-peptide linker should be taken into consideration [34,35].

In previous studies [35-37], the length and composition of the linkers that have been used to link VH and VL on bivalent single-chain antibody often impact stability and function. Linkers may be too short to fold correctly by intermolecular static influence or be too long to ameliorate the immunogenicity of antibodies. To satisfy these requirements, several design strategies have been developed. One approach is to use the flexible Glycine rich sequences (G_4_S)n as tethers. Linkers comprising repeats of G_4_S have been used to construct bivalent single-chain antibodies targeting colorectal cancer with linkers of 5-15 amino acids [18,36]. With a 5 amino acid linker, immune reactivity was unsatisfactory, possibly because the linker was too short to provide an effective distance for the two antigen-binding sites, which affected the stability of the cross-linked protein. The linker with 15 amino acids tended to fold correctly and retained the bivalent single-chain antibody's affinity and capacity. It has long been noted that sufficient flexibility and length for VH and VL domains are achieved by assembling them in the natural Fv orientation to form a monovalent antigen-binding site, which is comparable to the Fab fragment of native antibodies. It has also been shown that the length and sequence of the linker peptide significantly affects scFv expression and stability [36].

It should be pointed out that the impact of linker length on the activity and affinity of engineered antibodies depends strongly on the distance between the N- and C-terminal of the VH domain [37]. A certain degree of flexibility in the linker is required for the functional cooperation of the two subunits. The goal of this study was to characterize novel scFvs and to quantify the impact of linker peptide on binding affinity. Using computer guided homology, scFvs with different linker peptides were proposed based upon the activity and the End-to-end distance. Our aim was to evaluate the impact of (G_4_S)n on the structure and function of VH and VL, and to find the relationship between VH/VL’s End-to-end distance and *n* (or BC) on bivalent single-chain antibodies targeting colorectal cancer. A multi-factor relationship model was established to evaluate VH and VL structural factors using the following formula: $r\left(n\right)=\sqrt{{\left[AB\left(n\right)-AB_{O}\right]}^{2}+{\left[CD\left(n\right)-CD_{O}\right]}^{2}}+{\left[BC\left(n\right)-BC_{\mathrm{st}}\right]}^{2}$. Based on simulated data and biological experiments, a linear relationship has been established between the immunoreactivity and *r*-values. The immunoreactivity declines as the *r*-value increases. Fusion protein structure is ideal when the *r* = 3. When the *n* value is 6, protein structure is least satisfactory. However, further exploration of this relationship is needed. Indeed, the expression level and activity of scFv depends largely on the length and sequence of linker. Thus, successful construction of a scFv depends on the selection of a linker that neither interferes with the folding and association of VH and VL domains nor reduces the stability and recognition abilities of the Fv molecule.

In summary, based on the databases of natural protein structures and their associated functions, we predicted the structure and function of fusion proteins by homology modeling and further conducted biological experiments to validate our calculations. Thus, a dual approach that incorporates molecular modeling and linker design of engineered antibodies with quantitative determination of antibody affinity is useful to optimize construction. Our approach provides not only a rationale for designing novel engineered antibodies using molecular modeling, but also provides new insight into quantifying antibody binding affinity, especially at low protein concentration. A combination of bioinformatics and genetic research may therefore be beneficial in exploring new agents for genetic engineering of antibodies.

## Abbreviations

scFv: Single-chain Fv antibody; MCS: Multi-cloning site; PCA: Principal component analysis.

## Competing interests

The author(s) declare that they have no competing interests.

## Authors' contributions

JH Zhang drafted the manuscript. ZG Shang participated in the design of the study and performed the statistical analysis. L Shi conceived of the study, and participated in its design and coordination and helped to draft the manuscript. All authors read and approved the final manuscript.

## References

[B1] Olafsen T Sirk SJ Betting DJ Kenanova VE Bauer KB Ladno W Raubitschek AA Timmerman JM Wu AM ImmunoPET imaging of B-cell lymphoma using 124I-anti-CD20 scFv dimers (diabodies) Protein Eng Des Sel 2010 23 243 249 10.1093/protein/gzp081 20053640 PMC2841542

[B2] Asano R Ikoma K Kawaguchi H Ishiyama Y Nakanishi T Umetsu M Hayashi H Katayose Y Unno M Kudo T Kumagai I Application of the Fc fusion format to generate tag-free bi-specific diabodies FEBS J 2010 277 477 487 10.1111/j.1742-4658.2009.07499.x 20015073

[B3] Fisher AC DeLisa MP Efficient isolation of soluble intracellular single-chain antibodies using the twin-arginine translocation machinery J Mol Biol 2009 385 299 311 10.1016/j.jmb.2008.10.051 18992254 PMC2612092

[B4] Geng SS Feng JN Li Y Sun Y Gu X Huang Y Wang Y Kang X Chang H Shen B Binding activity difference of anti-CD20 scFv-Fc fusion protein derived from variable domain exchange Cell Mol Immunol 2006 3 439 443 17257497

[B5] Muller D Karle A Meissburger B Höfig I Stork R Kontermann RE Improved pharmacokinetics of recombinant bispecific antibody molecules by fusion to human serum albumin J Biol Chem 2007 282 12650 12260 10.1074/jbc.M700820200 17347147

[B6] Stone E Hirama T Tanha J Tong-Sevinc H Li S MacKenzie CR Zhang J The assembly of single domain antibodies into bispecific decavalent molecules J Immunol Methods 2007 318 88 94 10.1016/j.jim.2006.10.006 17141798

[B7] Guo JQ Li QM Zhou JY Zhang GP Yang YY Xing GX Zhao D You SY Zhang CY Efficient recovery of the functional IP10-scFv fusion protein from inclusion bodies with an on-column refolding system Protein Expr Purif 2006 45 168 174 10.1016/j.pep.2005.05.016 16125970

[B8] Shan DM Press OW Tsu TT Hayden MS Ledbetter JA Characterization of scFv-Ig constructs generated from the anti-CD20 mAb 1F5 using linker peptides of varying lengths J Immunol 1999 162 6589 6595 10352275

[B9] Shen Z Yan H Zhang Y Mernaugh RL Zeng X Engineering peptide linkers for scFv immunosensors Anal Chem 2008 80 1910 1917 10.1021/ac7018624 18290668 PMC2505110

[B10] James BG Leisha SM Frank MR Effect of linker sequence on the stability of circularly permtuted variants of ribonuclease T1 Bioorganic Chemistry 2003 31 412 424 10.1016/S0045-2068(03)00079-8 12941293

[B11] Arcangeli C Cantale C Galeffi P Gianese G Paparcone R Rosato V Understanding structural/functional properties of immunoconjugates for cancer therapy by computational approaches J Biomol Struct Dyn 2008 26 35 48 10.1080/07391102.2008.10507221 18533724

[B12] Wajanarogana S Prasomrothanakul T Udomsangpetch R Tungpradabkul S Construction of a human functional single-chain variable fragment (scFv) antibody recognizing the malaria parasite Plasmodium falciparum Biotechnol Appl Biochem 2006 44 55 61 10.1042/BA20050144 16398642

[B13] Clark KR Walsh ST Crystal structure of a 3B3 variant–a broadly neutralizing HIV-1 scFv antibody Protein Sci 2009 18 2429 2441 10.1002/pro.255 19785005 PMC2821263

[B14] Kamphausen S Holtge N Wirsching F Morys-Wortmann C Riester D Goetz R Thürk M Schwienhorst A Genetic algorithm for the design of molecules with desired properties J Comput Aided Mol Des 2002 16 551 567 10.1023/A:1021928016359 12602950

[B15] Hajduk PJ Huth JR Tse C Predicting protein druggability Drug Discov Today 2005 10 1675 1682 10.1016/S1359-6446(05)03624-X 16376828

[B16] Jiang Z Zhou Y Using bioinformatics for drug target identification from the genome Am J Pharmacogenomics 2005 5 387 396 10.2165/00129785-200505060-00005 16336003

[B17] Kim GB Wang Z Liu YY Stavrou S Mathias A Goodwin K.J, Thomas JM, Neville DM: A fold-back single-chain diabody format enhances the bioactivity of an anti-monkey CD3 recombinant diphtheria toxin-based immunotoxin Protein Eng Des Sel 2007 20 425 432 17693455 10.1093/protein/gzm040

[B18] Fang J Jin HB Song JD Construction, expression and tumor targeting of a single-chain Fv against human colorectal carcinoma World J Gastroenterol 2003 9 726 730 12679920 10.3748/wjg.v9.i4.726 PMC4611438

[B19] Arnold K Bordoli L Kopp J Schwede T The SWISS-MODEL Workspace: A web-based environment for protein structure homology modelling Bioinformatics 2006 22 195 201 10.1093/bioinformatics/bti770 16301204

[B20] Schwede T Kopp J Guex N Peitsch MC SWISS-MODEL: an automated protein homology-modeling server Nucleic Acids Res 2003 31 3381 3385 10.1093/nar/gkg520 12824332 PMC168927

[B21] Guex N Peitsch MC SWISS-MODEL and the Swiss-PdbViewer: An environment for comparative protein modelling Electrophoresis 1997 18 2714 2723 10.1002/elps.1150181505 9504803

[B22] McGuffin LJ Benchmarking consensus model quality assessment for protein fold recognition BMC Bioinforma 2007 8 345 10.1186/1471-2105-8-345 PMC2048972 17877795

[B23] Pawlowski M Gajda MJ Matlak R Bujnicki JM MetaMQAP: A meta-server for the quality assessment of protein models BMC Bioinforma 2008 9 403 10.1186/1471-2105-9-403 PMC2573893 18823532

[B24] Chen SC Chen TH Retrieval of 3D protein structures[C] 2002 New York: ICIP: Soifer Proceedings of the 2002 International Conference on Image Processing (ICIP 2002) 933 936

[B25] Szantai-Kis C Kovesdi I Eros D Nanhegyi P Ullrich A Orfi L Prediction oriented QSAR modelling of EGFR inhibition Curr Med Chem 2006 13 277 287 10.2174/092986706775476098 16475937

[B26] Le Gall F Reusch U Little M Kipriyanov Effect of linker sequences between the antibody variable domains on the formation, stability and biological activity of a bispecific tandem diabody Protein Eng Des Sel 2004 17 357 366 10.1093/protein/gzh039 15126676

[B27] Taylor WR Protein structure comparison using iterated double dynamic programming Protein Sci 1999 8 654 665 10091668 10.1110/ps.8.3.654 PMC2144286

[B28] Chen Q Zhou MQ Suo Q Wang Y Protein three-dimensional space unified coordinate system established Journal of Northwest University (Natural Science Edition) 2007 37 205 207

[B29] Tranchant I Hervé AC Carlisle S Lowe P Slevin CJ Forssten C Dilleen J Bhalla R Williams DE Tabor AB Hailes HC Design and synthesis of ferrocene probe molecules for detection by electrochemical methods Bioconjug Chem 2006 17 1256 1264 10.1021/bc060038m 16984136

[B30] Bello-Rivero I Torrez-Ruiz Y Blanco-Garcés E Pentón-Rol G Fernández-Batista O Javier-González L Gerónimo-Perez H López-Saura P Construction, purification, and characterization of a chimeric TH1 antagonist BMC Biotechnol 2006 6 25 10.1186/1472-6750-6-25 16716222 PMC1481661

[B31] Shibata K Maruyama-Takahashi K Yamasaki M Hirayama N G-CSF receptor-binding cyclic peptides designed with artificial amino-acid linkers Biochem Biophys Res Commun 2006 341 483 488 10.1016/j.bbrc.2005.12.204 16427611

[B32] Zou BJ Zhang Q Liang LM Similarity Comparison of Protein Structures via Protein Space Partition in Spherical Polar Coordinates Journal of Cmputer-aided Design & Computer Graphics 2009 21 205 207

[B33] Zhang JH Yun J Shang ZG Zhang XH Pan BR Design and optimization of a linker for fusion protein construction Prog Nat Sci 2009 19 1197 1200 10.1016/j.pnsc.2008.12.007

[B34] Fang M Jiang X Yang Z Yu XH Yin CC Li H Zhao R Zhang Z Lin Q Huang HL Effects of inter-peptide linkers to the biological activities of bispecific antibodies Chin Sci Bull 2003 48 1912 1918

[B35] Ying L Chen JH Zhang XG Research Progress in the Linker of Fusion Protein Biotechnology 2008 18 92 94

[B36] Yan DD Fang J Song JD Construction and Expression of Bivalent Single-chain Antibodies with Different Linker Sequence against Human Colorectal Carcinoma Chinese Journal of Cell Biology 2007 29 272 276

[B37] Gu X Jia XL Feng JN Shen BF Huang Y Geng SS Sun YX Wang YG Li Y Long M Molecular Modeling and Affinity Determination of scFv Antibody: Proper Linker Peptide Enhances Its Activity Ann Biomed Eng 2010 38 537 549 10.1007/s10439-009-9810-2 19816775

